# Amlexanox Suppresses Osteoclastogenesis and Prevents Ovariectomy-Induced Bone Loss

**DOI:** 10.1038/srep13575

**Published:** 2015-09-04

**Authors:** Yong Zhang, Hanfeng Guan, Jing Li, Zhong Fang, Wenjian Chen, Feng Li

**Affiliations:** 1 From the Department of Orthopedics, Tongji Hospital, Tongji Medical College, Huazhong University of Science and Technology, 430030, Wuhan, P.R.China

## Abstract

The activity of protein kinases IKK-ε and TANK-binding kinase 1 (TBK1) has been shown to be associated with inflammatory diseases. As an inhibitor of IKK-ε and TBK1, amlexanox is an anti-inflammatory, anti-allergic, immunomodulator and used for treatment of ulcer, allergic rhinitis and asthma in clinic. We hypothesized that amlexanox may be used for treatment of osteoclast-related diseases which frequently associated with a low grade of systemic inflammation. In this study, we investigated the effects of amlexanox on RANKL-induced osteoclastogenesis *in vitro* and ovariectomy-mediated bone loss *in vivo*. In primary bone marrow derived macrophages (BMMs), amlexanox inhibited osteoclast formation and bone resorption. At the molecular level, amlexanox suppressed RANKL-induced activation of nuclear factor-κB (NF-κB), mitogen-activated protein kinase (MAPKs), c-Fos and NFATc1. Amlexanox decreased the expression of osteoclast-specific genes, including TRAP, MMP9, Cathepsin K and NFATc1. Moreover, amlexanox enhanced osteoblast differentiation of BMSCs. In ovariectomized (OVX) mouse model, amlexanox prevented OVX-induced bone loss by suppressing osteoclast activity. Taken together, our results demonstrate that amlexanox suppresses osteoclastogenesis and prevents OVX-induced bone loss. Therefore, amlexanox may be considered as a new therapeutic candidate for osteoclast-related diseases, such as osteoporosis and rheumatoid arthritis.

Bone is a dynamic organ that continuously undergoes remodeling involving osteoclastic bone resorption and osteoblastic bone formation^1^. Bone remodeling is strictly modulated by various factors including hormones, cytokines/chemokines, and biomechanical external stimuli. Osteoclasts are bone-resorbing multinucleated giant cells differentiated from monocyte-macrophage lineage precursor cells. The differentiation of osteoclast is induced by two crucial cytokines: macrophage colony-stimulating factor (M-CSF) and receptor activator of nuclear factor-κB (NF-κB) ligand (RANKL). M-CSF is important for the proliferation and survival of osteoclast precursor cells, and the constitutive expression of RANK^2^. RANKL is a cytokine of the tumor necrosis factor (TNF) family that stimulates the differentiation of osteoclast precursor cells into mature osteoclasts^3^,^4^. RANKL binds to its receptor RANK on the surface of osteoclast precursor cells and induces the recruitment and accumulation of the adaptor proteins TNF receptor-associated factors (TRAFs) especially TRAF6. The formation of the RANKL-RANK-TRAF6 complex subsequently results in the activation of NF-κB, MAPKs (ERK, JNK, and p38) signaling pathways, c-Fos, and nuclear factor of activated T cells cytoplasmic 1 (NFATc1). The activation of these transcription factors directly stimulates the expression of a number of osteoclastogenesis-specific genes, including TRAP, MMP-9, NFATc1 and cathepsin K, leading to osteoclast differentiation and bone resorption^5^,^6^,^7^,^8^.

An imbalance of bone remodeling often results from excessive resorption by osteoclasts, as seen in osteoclast-related disorders such as postmenopausal osteoporosis, rheumatoid arthritis (RA), periodontitis, Paget’s disease, and cancer metastasis to bone^9^,^10^,^11^. Therefore, inhibition of the genesis and activity of osteoclasts is one of the most effective strategy for the prevention and treatment of osteoclast-related diseases. Osteoclast-related disorders are associated with inflammation and increased production of pro-inflammatory cytokines^9^,^10^,^11^,^12^,^13^,^14^. Especially, postmenopausal osteoporosis, a condition affecting approximately 30% of all postmenopausal women in the western world^15^, has been characterized by excessive bone loss associated with a low grade of systemic inflammation after estrogen withdrawal^16^. Moreover, telomerase-deficient mice, a model for aging, exhibit osteoporosis due to defects in osteoblasts and increased osteoclastogenesis caused by inflammatory microenvironment. In the long bone of the aging mouse, tumor necrosis factor (TNF), interleukin 6 (IL-6), and NF-κB signaling were among the most overrepresented pathways^17^. The inflammatory cytokines, including TNF, IL-1 and IL-6, promote osteoclastogenesis and bone resorption through multiple mechanisms including increase of the production of macrophage-colony stimulating factor (M-CSF) and RANKL^13^,^18^, therefore are also called osteolytic cytokines^9^.

Recent evidence indicates that increased activity of protein kinases IKK-ε and TANK-binding kinase 1 (TBK1) is associated with the pathology of inflammatory diseases such as rheumatoid arthritis (RA) and Paget’s disease^19^,^20^,^21^,^22^,^23^,^24^. For example, IKK-ε is constitutively expressed and phosphorylated in synovial intimal lining of RA tissue, leading to uncontrolled IRF3-driven production of proinflammatory mediators, such as IFN-β, chemokines and matrix metalloproteinases^19^. A major contribution of IKK-ε to the pathogenesis of RA is further supported by the finding that IKK-ε deficient mice show less synovial inflammation in a passive K/BxN arthritis model owing to lower expression of inflammatory mediators^20^. In addition, IKK-ε single nucleotide polymorphisms have been linked to the early stages of RA^22^. Furthermore, Hammaker *et al*. indicates that as it pertains to IFN- γ induced protein 10 kDa (IP-10), TBK1 might be more relevant than IKK-ε in the pathogenesis of RA and TBK1 is a potential therapeutic target in RA^23^. Moreover, TBK1 mediates critical effects of measles virus nucleocapsid protein (MVNP) on pagetic osteoclast formation^24^.

Amlexanox is an anti-inflammatory, anti-allergic immunomodulator used for the treatment of recurrent aphthous ulcers, allergic rhinitis and asthma^25^,^26^. Its mechanism of action might involve inhibition of inflammation by reducing the release of histamine and Leukotrienes. Recently, a study indicates that amlexanox is an inhibitor of TBK1 and IKK-ε, and that administration of amlexanox to obese mice produces reversible weight loss and restores insulin sensitivity, reduces inflammation and attenuates hepatic steatosis without affecting food intake^27^. Therefore, amlexanox may be a potential therapeutic agent for treatment of inflammatory diseases.

Whether amlexanox can be used for treatment of osteoclast-related diseases are not known. In the present study, we investigated the effects of amlexanox on RANKL-induced osteoclastogenesis *in vitro* and ovariectomy-induced bone loss *in vivo*, as well as osteoblast differentiation of BMSCs, and the underlying molecular mechanisms of these effects.

## Results

### Amlexanox inhibits RANKL-induced osteoclastogenesis *in vitro*

**Figure 1:**
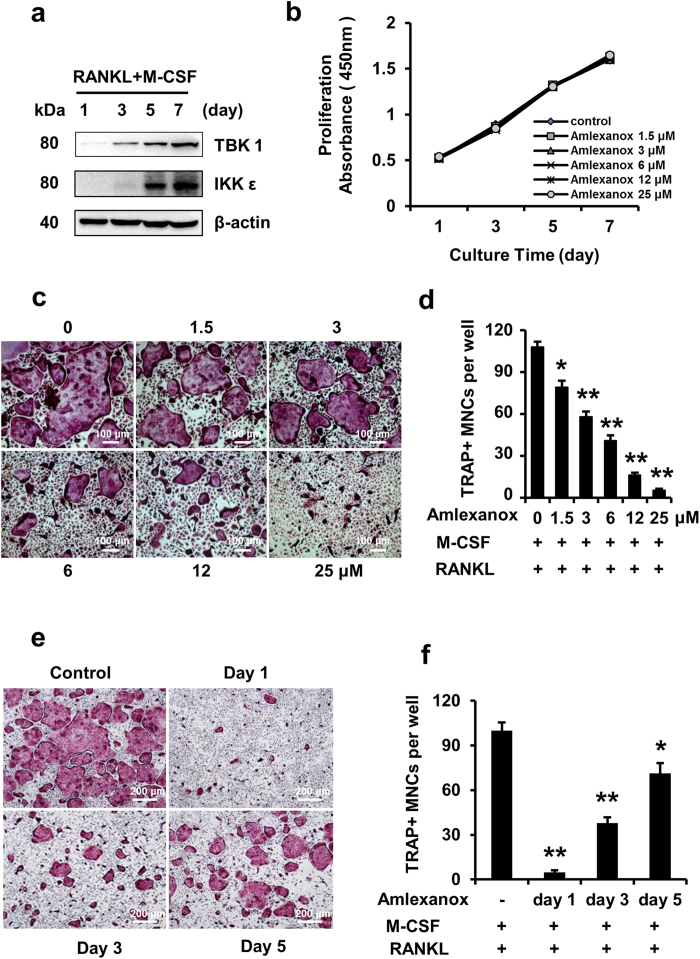
Amlexanox inhibits RANKL-induced osteoclastogenesis. (**a**) BMMs were seeded on day 0. RANKL was added on day 1 and every 2 days thereafter. Cells were collected for analysis of protein expression of TBK1 and IKK-ε. ACTB was used as a loading control. (**b**) Amlexanox has little effect on proliferation of BMMs. BMMs (5 × 10^3^ cells/well) were cultured with M-CSF (30 ng/ml), amlexanox was added at different concentrations (0, 1.5, 3, 6, 12, 25 μM) every 2 days for 7 days. Cell proliferation was assessed by Cell Counting Kit-8. Data was presented as mean ± SD of 3 independent experiments. (**c,d**) Amlexanox inhibits osteoclast formation in a dose-dependent manner. BMMs (1 × 10^4^ cells/well) were treated with different concentrations of amlexanox every 2 days in the presence of RANKL (50 ng/mL) and M-CSF (30 ng/ml) for 7 days. Then cells were fixed and stained for TRAP assay. TRAP-positive multinucleated osteoclasts (>=3 nuclei) were counted. Data was presented as mean ± SD of 3 independent experiments. Scale bar represents 100 μm. (**e,f**) BMMs (1 × 10^4^ cells/well) were cultured in the presence of RANKL (50 ng/mL) and M-CSF (30 ng/ml) for 7 days and treated with 25 μM amlexanox at the indicated times. Subsequently, cells were photographed and TRAP-positive multinucleated osteoclasts (>=3 nuclei) were counted. Data was presented as mean ± SD of at least 3 independent experiments. *P < 0.05, **P < 0.01 versus vehicle. Scale bar represents 200 μm (**e**).

We examined expression of TBK1 and IKK-ε during RANKL-induced osteoclastogenesis. Upon RANKL treatment, the protein expression of TBK1 and IKK-ε increased significantly and peaked at the last (7^th^) day (Fig. 1a). We then asked whether inhibition of TBK1 and IKK-ε by amlexanox had impact on osteoclastogenesis. We first evaluated the potential toxicity of amlexanox by measuring the proliferation of BMMs treated with different concentrations of amlexanox by Cell Counting Kit-8. As shown in Fig. 1b, amlexanox did not significantly affect the proliferation of BMMs. Next, BMMs were treated with different concentrations of amlexanox every 2 days in the presence of RANKL (50 ng/mL) and M-CSF (30 ng/ml) for 7 days. As shown in Fig. 1c,d, amlexanox inhibited osteoclast formation in a dose-dependent manner, with a half maximal inhibitory concentration (IC_50_) of 3 to 6 μM. Osteoclastogenesis is a multistep process consisting of proliferation, differentiation, cell fusion, and multinucleation^28^. To determine at which stage amlexanox blocked osteoclastogenesis, we added amlexanox at different time points during RANKL - induced osteoclastogenesis (Fig. 1e,f). Our results demonstrated that administration of amlexanox on the first day strongly inhibited RANKL-induced osteoclastogenesis. Amlexanox added at later stages also inhibited osteoclast formation, though to a lesser degree. These findings show that the inhibition of RANKL-induced osteoclastogenesis by amlexanox is not stage-specific, amlexanox might influenced pathways important for both early and late stages of osteoclast formation.

### Amlexanox inhibits osteoclast function.

**Figure 2:**
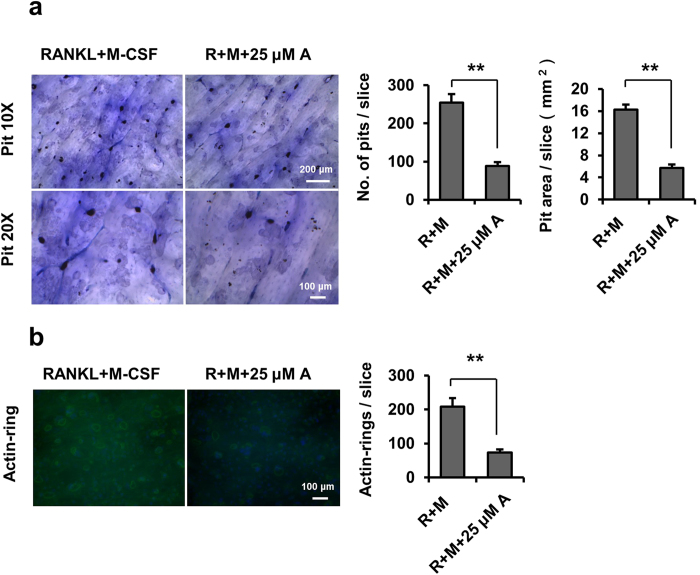
Amlexanox inhibits osteoclast function. (**a**) Amlexanox inhibits bone resorption of mouse osteoclasts on bone slices. The osteoclast preparation recovered from a collagen-gel culture was placed on bone slices, and treated with or without 25 μM amlexanox for 3 days. After removal of cells, bone slices were stained with toluidine blue. The number of resorption pits was counted. The area of resorption pits were measured. Data represent as mean ± SD for three bone slices. *P < 0.05; **P < 0.01. (**b**) Amlexanox disrupts the actin ring-formation by mature osteoclasts on bone slices. After culturing for 48 h, actin ring formation staining were performed, and examined by fluorescence microscopy. Osteoclasts with actin rings were counted. Data represent as mean ± SD. *P < 0.05; **P < 0.01.

To further examine whether amlexanox inhibited osteoclast function, we performed pit formation and actin ring formation assays using mature osteoclasts recovered from collagen-gel culture. Mature osteoclasts were seeded on bone slices in the presence of M-CSF and RANKL, and treated with or without 25 μM amlexanox for 3 days. Amlexanox significantly inhibited the pit formation activity of osteoclasts (Fig. 2a). We next examined the actin ring formation, which is essential for osteoclast attachment and bone resorption. Amlexanox markedly disrupted actin ring formation of osteoclasts (Fig. 2b). These results indicate that amlexanox inhibits the function of mature osteoclasts.

### Amlexanox inhibits RANKL-induced NF-κB and MAPKs activation

**Figure 3:**
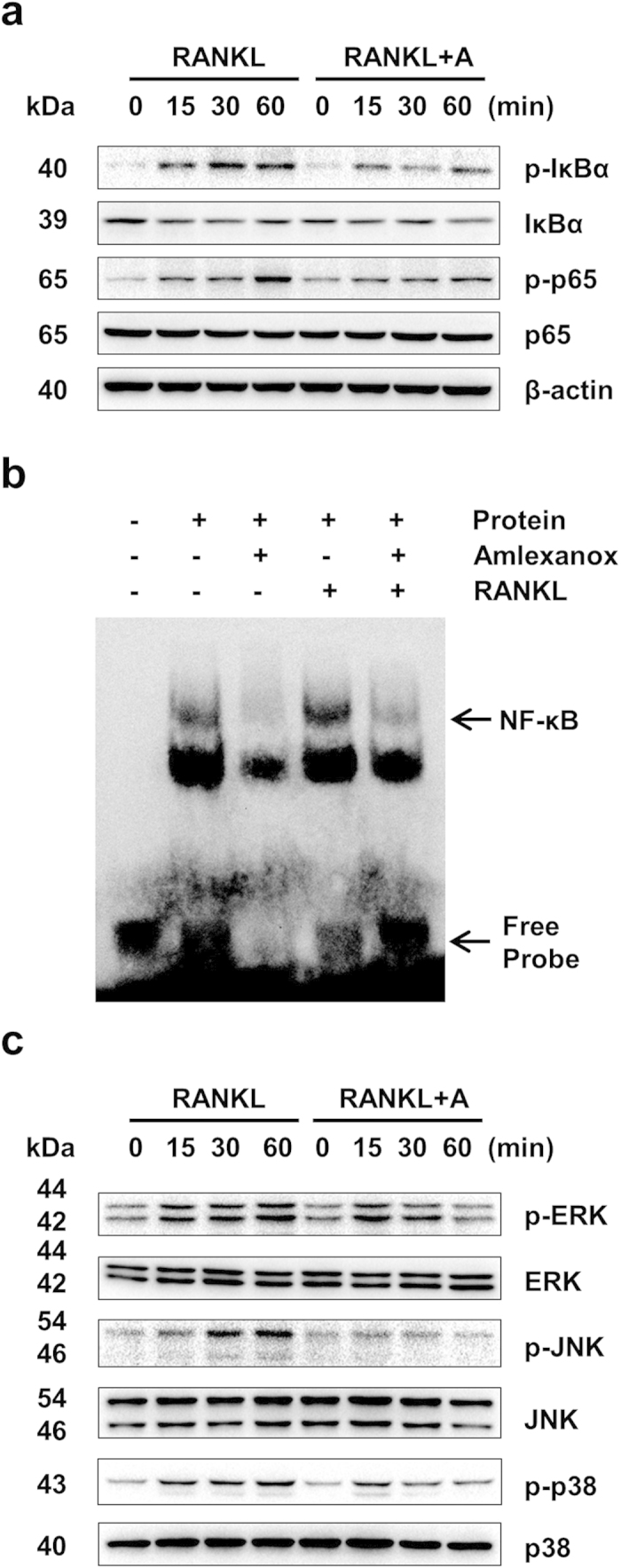
Amlexanox inhibits RANKL-induced NF-κB activation and the phosphorylation of MAPKs. (**a**) Amlexanox inhibits RANKL-induced phosphorylation of NF-κB/p65. We starved BMMs with 0.5% FBS in α-MEM for 12 h before treatment. BMMs were then pretreated with or without amlexanox (25 μM) for 1 h and then stimulated with RANKL (100 ng/mL) for the indicated times. The cell lysates were extracted for immunoblotting with the indicated antibodies. (**b**) Amlexanox suppresses RANKL-induced NF-κB DNA-binding activity. BMMs were pretreated with or without 25 μM amlexanox for 1 h and then stimulated with or without RANKL (100 ng/mL) for 30 min. Then the nuclear protein was prepared and subjected to EMSA. The Arrows indicate the free probe and the probe-NF-κB complex, respectively. (**c**) Amlexanox inhibits RANKL-induced phosphorylation of ERK, JNK and p38. We starved BMMs with 0.5% FBS in α-MEM for 12 h before treatment. BMMs were then pretreated with or without amlexanox (25 μM) for 1 h and then stimulated with RANKL (100 ng/mL) for the indicated times. The cell lysates were extracted for immunoblotting with the indicated antibodies.

Activation of NF-κB and MAPKs plays pivotal roles in osteoclast differentiation and function^9^,^29^,^30^,^31^,^32^,^33^. To investigate whether amlexanox suppress osteoclast differentiation through repression of NF-κB pathway, we examined NF-κB activation in BMMs using two methods. First, using western blot assays, we demonstrated that amlexanox could suppress RANKL-induced phosphorylation and degradation of IκBa, and the phosphorylation of NF-κB p65 (Fig. 3a). Second, we showed that amlexanox inhibited RANKL-induced NF-κB DNA-binding activity by EMSA assays (Fig. 3b). Together, our results indicate that amlexanox can inhibit RANKL-induced NF-κB activation. We next examined the phosphorylation of MAPKs (ERK, JNK, and p38) in BMMs by Western blot assays. As shown in Fig. 3c, amlexanox inhibited RANKL-induced phosphorylation of ERK, JNK and p38.

### Amlexanox inhibits RANKL-induced c-Fos and NFATc1 expression

**Figure 4:**
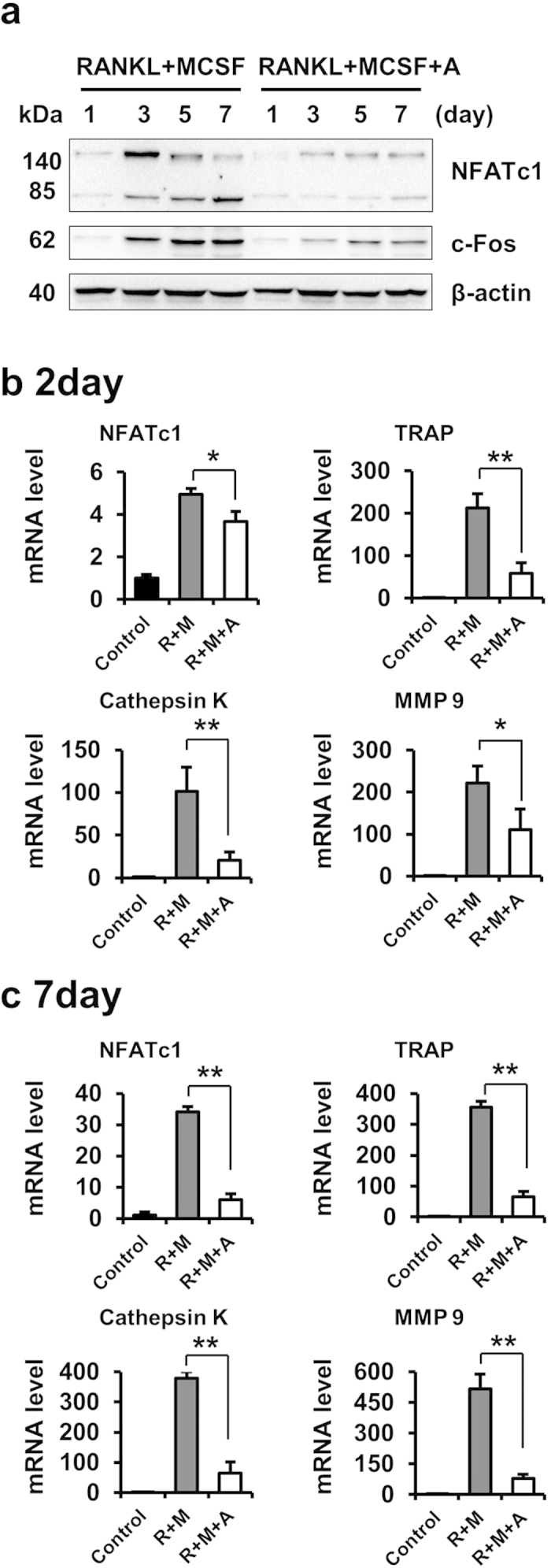
Amlexanox suppresses expression of c-Fos/NFATc1 and osteoclast maker genes. (**a**) Amlexanox inhibits RANKL-induced protein expression of c-Fos and NFATc1. BMMs were treated with or without amlexanox (25 μM) in the presence of RANKL (50 ng/mL) and M-CSF (30 ng/ml) for 7 days. Protein expression levels of c-Fos and NFATc1 were examined by western blot analysis at the indicated times. (**b,c**) Amlexanox suppresses RANKL-induced mRNA expression of TRAP, NFATc1, Cathepsin K, and MMP9. BMMs were treated with or without amlexanox (25 μM) in the presence of RANKL (50 ng/mL) and M-CSF (30 ng/ml) for 2 or 7 days. Total RNA was isolated and analyzed by quantitative real-time RT-PCR. Data represent as mean ± SD. *P < 0.05; **P < 0.01.

NFATc1 is a master transcription factor for osteoclast differentiation and function^8^,^32^. The transcription factor c-Fos acts as a key upstream activator of NFATc1 during osteoclastogenesis^33^,^34^. To determine whether amlexanox inhibits the expression of c-Fos and NFATc1 during RANKL-induced osteoclast differentiation, we examined the expression of c-Fos and NFATc1 by Western blot analysis. As shown in Fig. 4a, the protein expression of c-Fos and NFATc1 were increased during RANKL-induced osteoclast differentiation, and amlexanox efficiently prevented the increase of expression of c-Fos and NFATc1 induced by RANKL.

### Amlexanox suppresses the expression of osteoclast maker genes

Osteoclastogenesis is affected by the expression of several osteoclast-specific genes, such as TRAP, MMP9, NFATc1 and Cathepsin K. All of these are target genes of NFATc1^28^. Because amlexanox inhibited the expression of c-Fos/NFATc1 during osteoclast differentiation, we next tested whether amlexanox suppressed the expression of these osteoclast marker genes. Our results indicate that amlexanox significantly inhibits the mRNA expression of TRAP, NFATc1, MMP 9, and Cathepsin K at both early- and late- stage of osteoclastogenesis (Fig. 4b,c).

### Amlexanox enhances osteoblast differentiation of BMSCs

We also examined the effect of amlexanox on osteoblastogenesis *in vitro* by alkaline phosphatase (ALP) staining and alizarin red staining. The ALP positive regions were significantly increased when BMSCs were treated with amlexanox, especially at 25 μM (Supplementary Fig. 1a). Moreover, amlexanox enhanced bone nodule formation of BMSCs (Supplementary Fig. 1b). These results show that amlexanox enhances osteoblast differentiation of BMSCs *in vitro*. NF-κB activation plays a negative role in osteoblast differentiation^35^,^36^. To investigate whether amlexanox enhanced osteoblast differentiation through repression of NF-κB pathway, we examined NF-κB/p65 activation during osteoblast differentiation of BMSCs. The results demonstrated that amlexanox could moderately suppress the degradation of IκBa and the phosphorylation of NF-κB /p65 (Supplementary Fig. 2a). MAPKs activation also plays an essential role in osteoblastogenesis^37^. We examined the phosphorylation of MEK and MAPKs (ERK, JNK, and p38) in BMSCs by Immunobloting. As shown in Supplementary Fig. 2b, amlexanox significantly promoted the phosphorylation of MEK , JNK and p38.

### Amlexanox prevents OVX - induced bone loss.

**Figure 5:**
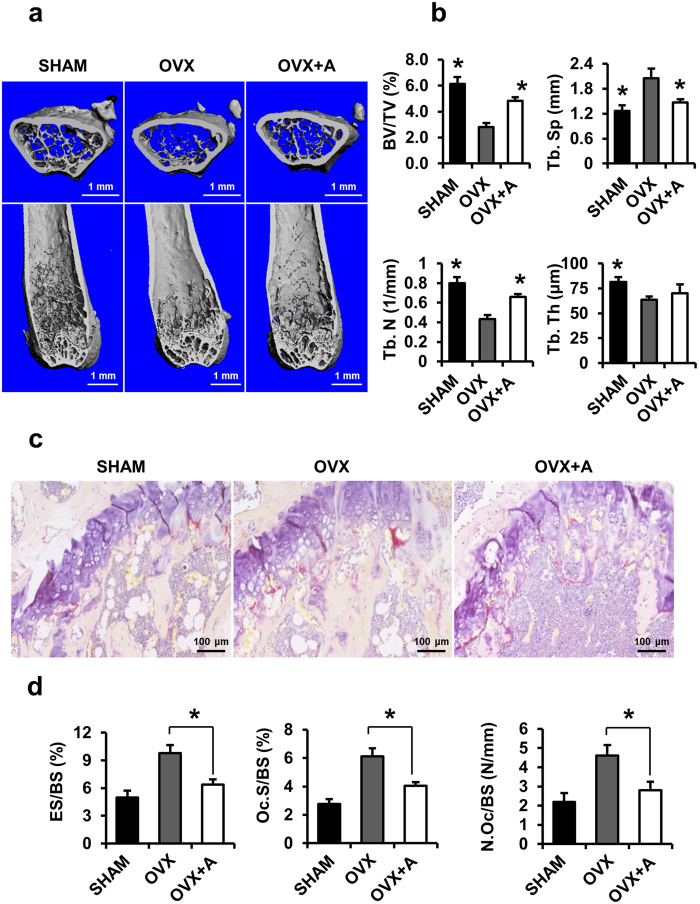
Amlexanox inhibits OVX-induced bone loss. (**a**) OVX mice were sacrificed after 8 weeks of amlexanox treatment. μ-CT images of the distal femurs from sham-operated group (SHAM), OVX and OVX+Amlexanox were obtained (top, axial view of the metaphyseal region; bottom, longitudinal view). (**b**) Histograms represent the parameters of three-dimensional trabecular structural of the distal femur: Trabecular bone volume/tissue volume (BV/TV), trabecular number (Tb. N), trabecular thickness (Tb. Th), and trabecular separation (Tb. Sp). Data represent as mean ± SD. n = 12. *P < 0.05; **P < 0.01 versus OVX. (**c**) Sections of the metaphyseal regions of the distal femurs from sham-operated group (SHAM), OVX and OVX+Amlexanox were performed TRAP staining. (**d**) For static histomorphometric analysis, femurs were fixed and then embedded in paraffin. The paraffin-embedded bone sections were stained for TRAP. Osteoclast surface/bone surface (Oc.S/BS, %), eroded surface/bone surface (ES/BS, %), and osteoclast number/bone surface (N.Oc/BS, N/mm) were measured. Data represent as mean ± SD. n = 12. *P < 0.05; **P < 0.01.

We next used the ovariectomized (OVX) mouse model to mimic menopause-induced bone loss in women^38^. The OVX mice showed marked atrophy and decreased wet weight of the uterus compared with the sham-operated mice (Supplementary Fig. 3a,b). Amlexanox (20 mg/kg) showed little effect on body weight over 8 weeks (Supplementary Fig. 3c), suggesting little toxicity of the small-molecule compound at the tested concentration, which is consistent with a previous study^27^. Micro-computed tomography (μ-CT) was used to analyze femurs from different groups of mice. The analysis of trabecular bone in distal femoral metaphyses demonstrated that BV/TV, Tb.N and Tb.Th in the OVX mice decreased dramatically, whereas Tb.Sp was significantly increased when compared with sham-operated group. Treatment of amlexanox (20 mg/kg) in OVX mice (OVX+Amlexanox) significantly inhibited the OVX-induced bone loss as measured in these parameters (Fig. 5a,b). To investigate whether amlexanox prevents bone loss through inhibition of osteoclastogenic activity *in vivo*, we performed TRAP staining on the femoral sections. The activity and size of osteoclasts in OVX mice increased markedly compared with sham-operated group. Treatment of OVX mice by amlexanox dramatically decreased the OVX-induced osteoclast activity (Fig. 5c,d). Histomorphometric analysis confirmed that Oc.S/BS, ES/BS, and N.Oc/BS strikingly increased in OVX mice compared with sham-operated group (Fig. 5d). These osteoclastic parameters were significantly decreased in OVX mice treated with amlexanox, as compared with the OVX mice (Fig. 5d).

**Figure 6:**
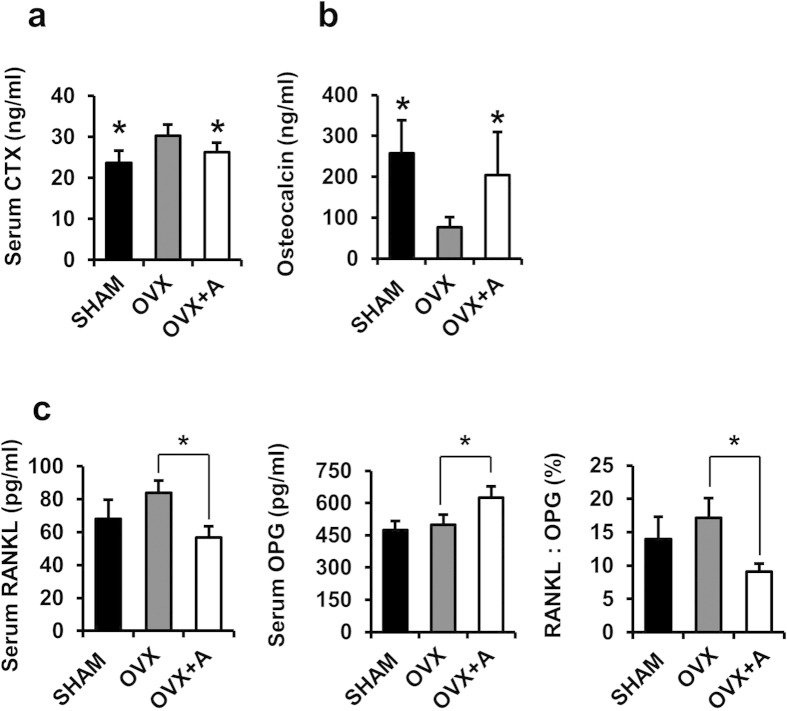
Serum levels of CTX-I, osteocalcin, RANKL and Osteoprotegerin (OPG) in mice treated with amlexanox. Mice were sham ovariectomized or ovariectomized as described, and treated with vehicle or amlexanox (20 mg/kg/d) for 8 weeks. Serum were collected before sacrifice. Serum levels of CTX-I (**a**) and osteocalcin (**b**) in SHAM, OVX and OVX+A mice were measured by ELISA. Data represent as mean ± SD. n = 12.*P < 0.05. (**c**) Serum levels of RANKL and OPG, and the RANKL: OPG ratio of animals was determined by ELISA. Data represent as mean ± SD. n = 12. *P < 0.05.

Moreover, the serum levels of type 1 collagen cross-linked C-terminal telopeptide (CTX-I), a bone resorption marker, were increased in OVX mice compared with sham-operated control mice, whereas amlexanox treatment significantly decreased the CTX-I levels induced by OVX (Fig. 6a). In addition, the serum levels of osteocalcin, a marker of bone turnover, were significantly increased in amlexanox-treated mice compared to that in OVX mice (Fig. 6b). Since the balance between RANKL and OPG produced by osteoblast lineage cells is critical for osteoclastogenesis and function, we examined the serum levels of RANKL and OPG by ELISA. In the OVX mice, serum levels of RANKL were increased, whereas amlexanox treatment obviously decreased the RANKL levels induced by OVX (Fig. 6c). The serum OPG levels in OVX mice was not significantly different from that of the sham-operated mice. However, amlexanox treatment markedly increased the serum OPG levels. As a result, the RANKL/OPG ratio was significantly decreased in OVX mice treated with amlexanox, as compared with the OVX mice (Fig. 6c).

## Discussion

In the present study, we investigated the effects of amlexanox on RANKL-induced osteoclastogenesis *in vitro* and ovariectomy-induced bone loss *in vivo*, as well as osteoblast differentiation of BMSCs. We believe that amlexanox attenuated OVX – induced bone loss through multiple mechanisms: first, amlexanox directly inhibited osteoclast formation and activity, which might be the most important mechanism; second, amlexanox promoted osteoblastogenesis from BMSCs *in vitro* and led to higher serum osteocalcin levels *in vivo*, suggesting that it might enhance bone formation; third, by regulating production of RANKL and OPG synthesized by osteoblast lineage cells and immune cells, amlexanox might inhibit osteoclastogenesis and bone resorption indirectly; fourth, amlexanox was shown to reduce serum concentrations of multiple osteolytic cytokines including IL-1α and TNF-α^27^, which might also lead to suppressed osteoclastogenesis.

At the molecular level, amlexanox inhibited multiple pathways downstream of RANKL, including MAPKs, NF-kB, NFATc1 and c-fos. The NF-κB signaling pathway is one of the essential pathways for osteoclast formation and activity^1^,^9^,^29^. We showed that amlexanox inhibited RANKL-induced activation of the NF-κB signaling pathway, as demonstrated by inhibition of phosphorylation of both IκBa and p65, and the DNA-binding activity of NF-κB. We believe NF-κB might be one of the most important downstream pathways that mediating the effects of amlexanox on osteoclastogenesis. The regulation of NF-κB by TBK1 or IKK-ε has long been a controversial topic^27^. A previous study showed TBK1 positively modulates RelA/p65 phosphorylation, which is in agreement with our finding that amlexanox represses phosphorylation of p65 through inhibition of TBK1^39^. We also demonstrated that amlexanox moderately suppressed the degradation of IκBa and the phosphorylation of NF-κB/p65 during osteoblast differentiation. Therefore, amlexanox might promote osteoblast differentiation partially through inhibition of NF-κB, which plays a negative role in osteoblast differentiation^35^,^36^. MAPKs activation also plays an essential role in both osteoclastogenesis and osteoblastogenesis^37^. We found that amlexanox inhibited MAPKs in RANKL – induced osteoclastogenesis; whereas, during osteoblast differentiation, amlexanox promoted activation of MAPKs. We believe that the modulation of MAPKs in cells of the two lineages might not be a primary effect of amlexanox. Further efforts are warranted to shed light on these issues.

We found that amlexanox promoted osteoblastogenesis, the mechanisms of action are not completely known, whether amlexanox influenced the activity of key signaling pathways in osteoblast differentiation such as Wnt/β-catenin and RUNX2 remains unknown. Future work is needed to elucidate the involved mechanisms.

In a previous study, amlexanox was shown to produce weight loss, improve insulin sensitivity and decreased steatosis in obese mice through its anti-inflammatory properties^27^. Therefore, it was proposed to be a promising therapeutic agent for diabetes and obesity. Diabetes and obesity shares similar inflammatory pathways with osteoclast-related disorders such as postmenopausal osteoporosis, rheumatoid arthritis (RA) and osteoarthritis. Moreover, diabetes and/or obesity are frequently co-exist with osteoporosis and osteoarthritis in senior patients. Considering our current results and the proven pharmacologic safety of amlexanox in patients, we believe it might be worthwhile to try to re-purpose amlexanox for these inflammatory - related conditions.

## Methods

### Reagents

Amlexanox was purchased from Sigma Aldrich (St Louis, MO, USA). Recombinant soluble human macrophage-colony stimulating factor (M-CSF) and mouse receptor activator of nuclear factor-κB ligand (RANKL) were obtained from Peprotech (Rocky Hill, NJ, USA). The cell counting kit-8 (CCK-8) was purchased from Dojindo (Kumamoto, Japan). The following antibodies were purchased from Cell Signaling Technology (Beverly, MA, USA): ERK (#9102), phospho-ERK (#4377), JNK (#9258), phospho-JNK (#4668), p38 (#8690), phospho-p38 (#4511), p65 (#8242), phospho-p65 (#3033), IKK-ε (#2690), TBK1 (#3013), IKKβ (#8943), IκBα (#4812), phospho-IκBα (#2859), NFATc1 (#8032). Anti-c-Fos was purchased from Abcam (Cambridge, MA, USA). The NF-κB probe was purchased from Beyotime (Shanghai, China). The TRAP staining kit and all other reagents were purchased from Sigma Aldrich.

### Animals

C57/BL6 female mice and Sprague-Dawley rats were purchased from the Experimental Animal Center of Tongji Medical College (Wuhan, China). Mice were housed at the animal care facility of Tongji Medical College at 25 °C with 12-hour light/dark cycles and were allowed free access to normal mice chow and water. The animal studies were approved by the Institutional Animal Research Committee of Tongji Medical College. The methods of animal experiments were carried out in accordance with protocols approved by the Institutional Animal Care and Use Committee.

### Cell cultures

We cultured primary bone marrow cells isolated from C57/BL6 mice as described^40^,^41^. Briefly, Bone marrow cells were isolated from 8-week-old C57/BL6 mice by flushing femurs and tibias with α-MEM and cultured in α-MEM with 10% FBS, 100 U/ml penicillin, 100 μg/ml streptomycin and M-CSF (30 ng/ml) overnight. Non-adherent cells were collected and further cultured in the presence of M-CSF (30 ng/ml) for 3 days. Floating cells were discarded and adherent cells were used as bone marrow-derived macrophages (BMMs). Preparation of mouse osteoclasts was carried out as described previously^42^,^43^. In brief, BMMs (4 × 10^4^ cells/well) were seeded on a 0.2% collagen-gel coated 12-well plate and induced by RANKL (100 ng/mL) and M-CSF (30 ng/mL) for 6 days. Then osteoclasts were recovered by treatment with 0.2% collagenase, suspended in a-MEM containing 10% FBS, and used for osteoclast function assays.

Rat bone marrow mesenchymal stem cells (BMSCs) were isolated from 4-week-old Sprague–Dawley rats (male or female 80–100 g) and expanded in accordance with published techniques^44^,^45^,^46^. The cells were maintained in expansion medium consisting of Dulbecco’s modified Eagle’s medium (DMEM)/F12, 10% FBS.

### *In vitro* osteoclastogenesis assay

For induction of osteoclastogenesis, BMMs were seeded at a density of 1 × 10^4^ cells/well in 96-well plates in the presence of RANKL (50 ng/mL) and M-CSF (30 ng/ml) for 7 days. The culture medium was replaced every 2 days. Osteoclasts were identified by Tartrate-resistant acid phosphatase (TRAP) staining. TRAP-positive multinucleated cells with **>= **3 nuclei were counted as osteoclasts. Three wells were assessed per treatment in three independent experiments.

### Cell proliferation assay

To examine cell proliferation, a Cell Counting Kit-8 was used according to the manufacturer’s instructions. BMMs were seeded at a density of 5 × 10^3^ cells/well in 96-well plates. After 24 hours, cells were treated with different concentrations of Amlexanox (0, 1.5, 3, 6, 12, 25 μM) every 2 days in the presence of M-CSF (30 ng/ml) for 7 days. After 1, 3, 5 and 7 days, the culture medium was replaced by the medium containing 10% CCK-8 and cells were incubated at 37 °C for an additional 2 h. The absorbance was then measured at a wavelength of 450 nm on an ELX800 absorbance microplate reader (Bio-Tek, Vermont, USA).

### Pit formation assays and actin ring formation assays

We performed pit formation assay as described previously^40^. Briefly, osteoclasts recovered from a collagen-gel culture were placed on FBS coated bovine cortical bone slices adapted for 96-well plates (IDS Nordic, Herlev, Denmark), and treated with or without 25 μM amlexanox in the presence of RANKL (100 ng/mL) and M-CSF (30 ng/mL) for additional 3 days. Then the bone slices were treated with 1 M NH_4_OH with sonication for 5 minutes and stained with 0.5% toluidine blue at room temperature for 1 minute. The images of resorption pits were captured through light microscopy. The area and number of resorption pits were measured and analyzed as previously described^40^. The actin ring formation assay was done as described previously^47^,^48^.

### Ovariectomized mouse model

Three-month-old female C57/BL6 mice were divided randomly into three groups (n = 12 mice per group): sham-operated mice (SHAM), ovariectomized (OVX) mice treated with vehicle (OVX), and OVX mice treated with amlexanox (OVX + Amlexanox). As described earlier^40^, ovariectomy was performed by removing the bilateral ovaries through a dorsal approach and sham surgery was performed by identifying the bilateral ovaries. One day after surgery, mice were injected intraperitoneally (i.p.) with amlexanox (20 mg/kg) or vehicle every day for 8 weeks. After 8 weeks, all mice were euthanized with excess amounts of pentobarbitone. After euthanasia, the femurs and tibias were collected.

### Micro-computed tomography (μ-CT) and histomorphometric analysis

μ-CT was performed on the left femurs. The distal femoral metaphysis was scanned with a μ-CT system (μ-CT50 Scanco Medical, Bassersdorf, Switzerland). Image acquisition was performed at 100 kV and 98 μA, with a resolution of 2048 × 2048 pixels and a voxel size of 10 μm. Bone volume/tissue volume (BV/TV), Trabecular number (Tb. N.), Trabecular thickness (Tb. Th.), Trabecular separation (Tb. Sp.) was quantitatively analyzed using a software compatible with the μ-CT system. Nomenclature and abbreviations of 3D-μCT parameters follow the recommendations of the American Society of Bone and Mineral Research^49^.

For static histomorphometric analyses, femurs were fixed in 4% paraformaldehyde and decalcification was performed with 10% EDTA for 2 weeks. The samples were then embedded in paraffin. The paraffin-embedded bone sections were stained for TRAP, and the number of osteoclasts was counted as previously described^40^,^50^,^51^. Osteoclast surface/bone surface (Oc.S/BS, %), eroded surface/bone surface (ES/BS, %), and osteoclast number/bone surface (N.Oc/BS, N/mm) were measured. Briefly, all measurements were restricted to the secondary spongiosa and confined to an area between 400 and 2000 μm distal to the growth plate-metaphyseal junction of the distal femur.

### Measurement of serum levels of CTX-I, osteocalcin, RANKL and OPG

Sera were collected from SHAM, OVX and OVX+Amlexanox mice before they were sacrificed after 8 – week treatment with amlexanox. Serum CTX-I levels were measured using a RatLaps EIA kit (IDS Nordic, Herlev, Denmark). Serum osteocalcin levels were measured using a Mouse Osteocalcin EIA kit (Biomedical Technologies). Serum RANKL and OPG levels were measured using Mouse RANKL and OPG ELISA kit (BOSTER, Wuhan, China).

### Electrophoretic mobility shift assay (EMSA)

BMMs were pre-treated with or without 25 μM amlexanox for 1 h and then stimulated with RANKL (100 ng/mL) or vehicle for 30 min, and the extraction of nuclear proteins was performed as described previously^40^,^52^. The DNA-binding activity of NF-κB was detected using a chemiluminescent EMSA kit (Pierce, USA). Briefly, nuclear extracts were incubated with the probe in reaction buffer (1×binding buffer, 2.5% glycerol, 5 mM MgCl_2_, 50 ng/μl poly (dI-dC), and 0.05% NP-40) for 30 min. Reactants were loaded onto a 6% native polyacrylamide gel and transferred onto a positively charged nylon membrane (Millipore, Billerica, MA, USA). The DNA was cross-linked by UV cross-linker. The biotin end labeled DNA was detected using a Streptavidin-HRP conjugate and a chemiluminescent substrate. The membrane was then exposed to ChemiDoc™ XRS+ System with Image Lab™ Software (Bio-Rad, CA, USA).

### Quantitative real-time reverse transcription-PCR

Quantitative real-time reverse transcription-PCR (qRT-PCR) was performed as described previously^52^,^53^. Briefly, total RNA was extracted from osteoclasts using TRIZOL (Invitrogen, Carlsbad, CA, USA). First-stranded cDNA was synthesized from 2 μg of total RNA by Easy Script First-Strand cDNA Synthesis Super Mix kit (TransGen Biotech, Beijing, China). Quantitative real-time RT-PCR was performed on CFX96 (Bio-Rad, CA, USA) using Power SYBR Green PCR Master Mix (TransGen Biotech, Beijing, China). All reactions were performed in triplicates, and target genes expression was normalized to the reference gene glyceraldehyde-3-phosphate dehydrogenase (GAPDH). The primers used for quantitative real-time RT-PCR are listed in Table 1.

### Western blot analysis

Immunoblot analysis was performed as described previously^52^,^53^. Cells were lysed using the protein extraction reagent RIPA (BOSTER, Wuhan, China) supplement with 1 mM PMSF. The protein concentration was determined using the BCA assay. An equivalent amount of protein were resolved by 10% SDS-PAGE gel and transferred to PVDF membranes (Millipore, Billerica, MA, USA). Subsequently, membranes were blocked and immunoblotted with individual antibodies. The membranes were washed and incubated with horseradish peroxidase–conjugated secondary antibodies (BOSTER, Wuhan, China, dilution 1:5000). The immunoreactive proteins were visualized using enhanced chemiluminescence (BOSTER, Wuhan, China). The protein bands were captured using ChemiDoc™ XRS+ System with Image Lab™ Software (BIO-RAD).

### Statistical analysis

All quantitative data were presented as means ± SD from three independent experiments. Student’s t-test was used for determining the significance of differences between two groups, whereas one-way ANOVA was used for multiple comparisons. The difference was considered statistically significant at P < 0.05.

## Additional Information

**How to cite this article**: Zhang, Y. *et al.* Amlexanox Suppresses Osteoclastogenesis and Prevents Ovariectomy-Induced Bone Loss. *Sci. Rep.* **5**, 13575; doi: 10.1038/srep13575 (2015).

## Supplementary Material

Supplementary Information

## Figures and Tables

**Table 1:** Oligonucleotides used for quantitative real-time RT-PCR.

Target mouse gene	Sequence	GenBank reference
gapdh	(F) 5′-CTCCCACTCTTCCACCTTCG-3′	NM008084
	(R) 5′-TTGCTGTAGCCGTATTCATT-3′	
trap	(F) 5′-TACCTGTGTGGACATGACC-3′	BC029644
	(R) 5′-CAGATCCATAGTGAAACCGC-3′	
cathepsin K	(F) 5′-TGTATAACGCCACGGCAAA-3′	X94444
	(R) 5′-GGTTCACATTATCACGGTCACA-3′	
nfatc1	(F) 5′-CAACGCCCTGACCACCGATAG-3′	AF239169
	(R) 5′-GGGAAGTCAGAAGTGGGTGGA-3′	
mmp-9	(F) 5′-TCCAGTACCAAGACAAAGCCTA-3′	X72795
	(R) 5′-TTGCACTGCACGGTTGAA-3′	

Note: Forward (F) and reverse (R) primers are listed.

gapdh = glyceraldehyde-3-phosphate dehydrogenase; trap = tartrate resistant acid phosphatase; nfatc1 = nuclear factor of activated T cells 1; mmp-9=matrix metalloproteinase-9.
