# Supplementary material for: Amlexanox Suppresses Osteoclastogenesis and Prevents Ovariectomy-Induced Bone Loss
Source: Sci Rep. 2015 Sep 4;5:13575. doi: 10.1038/srep13575 (PMC4559750; doi:10.1038/srep13575)
Supplement: Supplementary Information [file srep13575-s1.pdf]

Supplementary Information

Amlexanox Suppresses Osteoclastogenesis and Prevents Ovariectomy-Induced Bone Loss

Yong Zhang[a], Hanfeng Guan[a], Jing Li, Zhong Fang, Wenjian Chen, Feng Li*

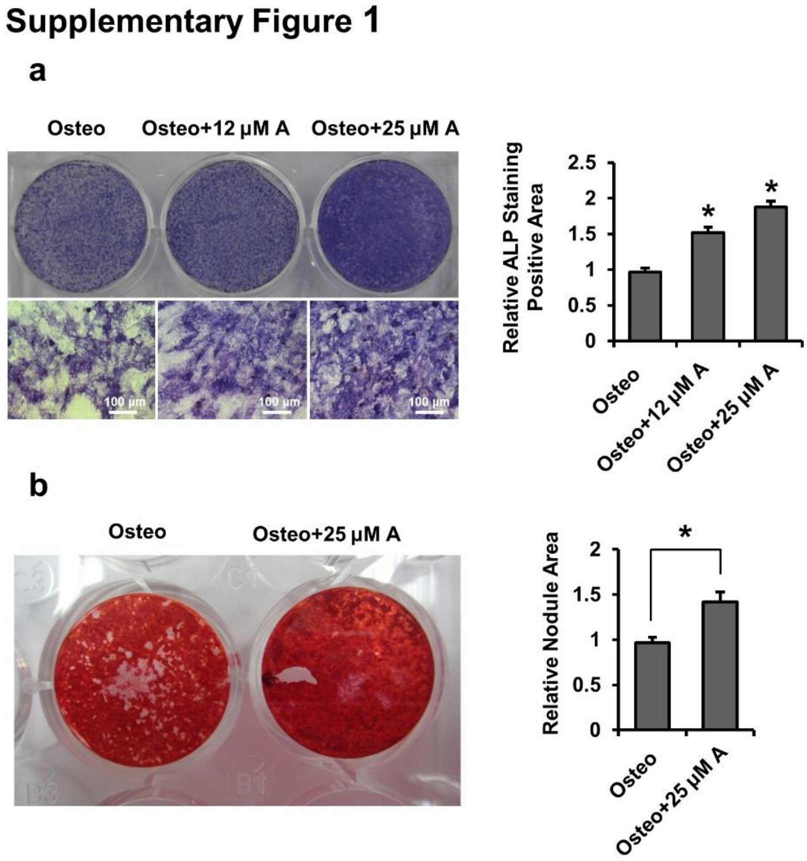

Supplementary Figure 1. Amlexanox enhances osteoblast differentiation of BMSCs. (a) BMSCs (1.0×10^5 cells/well) were incubated with SD Rat Mesenchymal Stem Cell Osteogenic Differentiation Medium, and were treated with different doses of amlexanox (0, 12, 25 μM) every 3 days for 10 days. The cells were fixed and stained with ALP solution at day 10 and photographed. (b) Amlexanox enhanced bone nodule formation. BMSCs (6×10^4 cells/well) were incubated with SD Rat Mesenchymal Stem

Cell Osteogenic Differentiation Medium, and cells were treated with or without amlexanox (25 μM) every 3 days for 21 days. The cells were fixed and Alizarin Red staining was performed at day 21. Positive staining regions and bone nodule formation were quantified using Image-Pro Plus. Data represent as mean ± SD.* $P < 0.05$.

Supplementary Figure 2

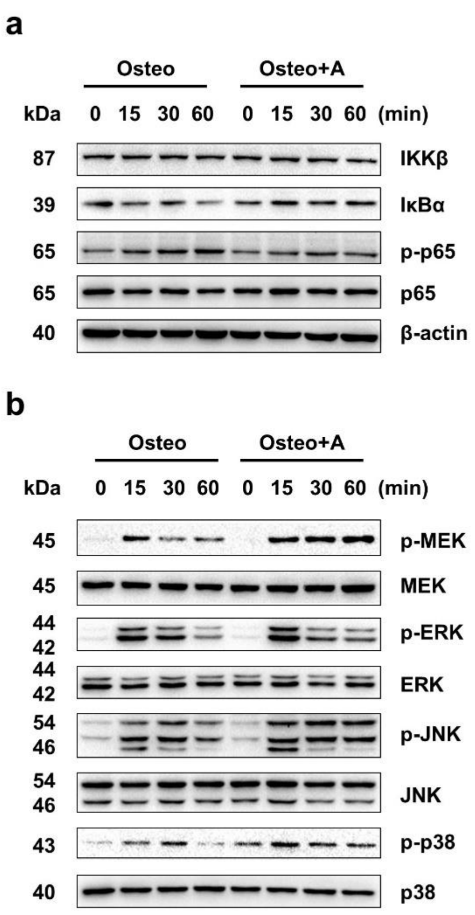

Supplementary Figure 2. Amlexanox inhibits NF-κB activation and promotes the activation of MAPKs. (a) Amlexanox inhibits NF-κB/p65 phosphorylation in BMSCs. **(b)** Amlexanox promotes the phosphorylation of MEK, JNK and p38 in BMSCs. BMSCs were pretreated with or without amlexanox (25 μM) for 1 h and then stimulated with SD Rat Mesenchymal Stem Cell Osteogenic Differentiation Medium for the

indicated times. The cell lysates were extracted for immunoblotting with the indicated antibodies.

Supplementary Figure 3

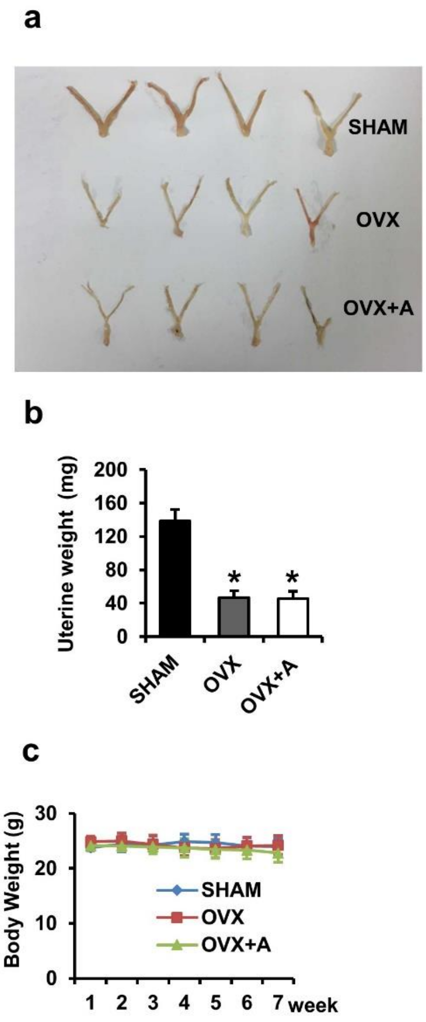

Supplementary Figure 3. Validation of the success of ovariectomy. (a, b) 8 weeks after ovariectomy, all mice were euthanized. The uteruses were phtographed (a) and weighed (b). (c) The body weight of the mice was measured. Amlexanox had little effect on mouse body weight at 20 mg/kg. Body weight was recorded every week. Data represent as mean ±SD. n=12. * P < 0.05 versus SHAM.

Supplementary methods

ALP and Alizarin red staining

For osteogenic differentiation, BMSCs were cultured with OriCell^{TM} Sprague-Dawley (SD) Rat Mesenchymal Stem Cell Osteogenic Differentiation Medium (Cyagen, Guangzhou, China). For ALP staining, cells were seeded at a density of 1.0×10^5 cells/well in 6-well culture plates pre-coated with Gelatin Solution. After 24 hours, 2 ml SD Rat Mesenchymal Stem Cell Osteogenic Differentiation Medium was added. BMSCs were then treated with different concentrations of amlexanox (0, 12, 25μM) every 3 days. The medium was changed every 3 days with fresh SD Rat Mesenchymal Stem Cell Osteogenic Differentiation Medium and amlexanox. After 10 days, ALP staining was performed using leukocyte alkaline phosphatase (LAP) kits (sigma, St. Louis, MO, USA). After discarding the media, cells were gently washed with 1×PBS for three times and fixed with 2ml 4 % paraformaldehyde for 15 min at room temperature. The cells were washed with deionized water, and then stained with Naphthol AS-MX Phosphate 30 min at 37 ℃ in a 5% CO_2 humidified incubator. The wells were then washed three times with PBS to remove excess dye. Images were observed and captured using a light microscope.

For Alizarin red staining, BMSCs were seeded at a density of 6×10^4 cells/well in 12-well culture plates pre-coated with Gelatin Solution. BMSCs were incubated with or without SD Rat Mesenchymal Stem Cell Osteogenic Differentiation Medium, and then cells were treated with Amlexanox (25μM) or vehicle every 3 days for 21 days. After

21 days, cells were rinsed with 1×PBS for two times and fixed with 1ml of 4% paraformaldehyde for 30 minutes at room temperature. Wells were rinsed with 1×PBS for two times and then cells were stained with 1ml alizarin red working solution for 3 to 5 minutes. The wells were then washed three times with PBS to remove excess dye. Cells were visualized and images were captured under light microscope.
